# Neuromuscular compartmentation of the subscapularis muscle and its clinical implication for botulinum neurotoxin injection

**DOI:** 10.1038/s41598-023-38406-0

**Published:** 2023-07-10

**Authors:** Tae-Hyeon Cho, Ju-Eun Hong, Hun-Mu Yang

**Affiliations:** 1 Department of Anatomy, College of Korean Medicine, Semyung University, Jecheon, Republic of Korea; 2 Translational Research Unit for Anatomy and Analgesia, Seoul, Republic of Korea; 3 Department of Biomedical Laboratory Science, College of Software and Digital Healthcare Convergence, Yonsei University MIRAE Campus, Wonju, Republic of Korea; 4 Translational Laboratory for Clinical Anatomy, Department of Anatomy, Yonsei University College of Medicine, Seoul, Republic of Korea

**Keywords:** Anatomy, Musculoskeletal system, Translational research

## Abstract

In this study, using immunohistochemistry with fresh cadavers, deliberate histological profiling was performed to determine which fibers are predominant within each compartment. To verify the fascial compartmentation of the SSC and elucidate its histological components of type I and II fibers using macroscopic, histological observation and cadaveric simulation for providing an anatomical reference of efficient injection of the BoNT into the SSC. Seven fixed and three fresh cadavers (six males and four females; mean age, 82.5 years) were used in this study. The dissected specimens revealed a distinct fascia demarcating the SSC into the superior and inferior compartments. The Sihler’s staining revealed that the upper and lower subscapular nerves (USN and LSN) innervated the SSC, with two territories distributed by each nerve, mostly corresponding to the superior and inferior compartments of the muscle, although there were some tiny communicating twigs between the USN and LSN. The immunohistochemical stain revealed the density of each type of fiber. Compared with the whole muscle area, the densities of the slow-twitch type I fibers were 22.26 ± 3.11% (mean ± SD) in the superior and 81.15 ± 0.76% in the inferior compartments, and the densities of the fast-twitch type II fiber were 77.74% ± 3.11% in the superior and 18.85 ± 0.76% in the inferior compartments. The compartments had different proportions of slow-fast muscle fibers, corresponding to the functional differences between the superior compartment as an early-onset internal rotator and the inferior compartment as a durable stabilizer of the glenohumeral joint.

## Introduction

The subscapularis muscle (SSC) muscle is the only cuff muscle to give strength in the direction of inwardly turning to the lesser tubercle (LT) of the humerus for the static and dynamic balance of the joint^1^. The pathological hyperactivity of the SSC results in an abnormally limited range of external rotation and abduction of the shoulder with consequent discomfort^2,3^. SSC spasticity is a frequent cause of shoulder pain, and botulinum neurotoxin (BoNT) is therapeutically used to reduce muscle tone by blocking the effect of acetylcholine on the motor end plate (MEP)^4^.

Clinical, pharmacological, and anatomical factors determine the effectiveness and safety of BoNT injection. The technical strategy for effective injection is dependent on the practical considerations of how efficiently the agents approach the intramuscular MEPs and which muscle fibers are mainly involved. Specifically, for effectiveness, a physician performing the injection should consider (1) the location of the MEPs to be affected, (2) physical resistance of the tissue, and (3) muscle fiber type to be attenuated.

A safe approach to the SSC is challenging because the muscle is located on the subscapular fossa facing the ribs of the trunk and is significantly close to the cardinal vessels and nerves. To overcome these anatomical obstacles, medial and lateral approaches toward the scapula have been recommended for safe needle advancement^5^. Either way, the upper subscapular nerve (USN) and lower subscapular nerve (LSN) have been anatomically studied based on the assumption that the MEP can be near the nerve distribution. A comprehensive verification of numerous MEPs with the whole muscle scale is challenging owing to its methodological mismatch with histological observation for a micrometer-scale positioning of the MEPs. Grasping the overall MEP position is only indirectly performed by observation on the intramuscular route and distribution pattern of the nerve. Two or three main intramuscular trunks are ramified from the USN and LSN and proceed at a distance. Hence, Warden et al. (2014) demarcated the SSC into two or three separate nervous territories^5^, and Cho et al.^4^ provided an intramuscular innervation of the SSC, showing two distinct nervous nervous territories using Sihler’s staining. Likewise, multiple injections into the SSC are more effective than single injections^5,6^.

The BoNT agent extends its influential range by spreading within the muscle; thus, physical resistance due to the histological condition can reduce the extent of the intramuscular spread of the BoNT^5,6^. For instance, a physical barrier of the fascial septum could reduce the spread of the agent^7^. We also carefully assumed the existence of the septum within the SSC in the previous study^4,5^; however, anatomical evidence was not provided until recently. Moreover, the spread of the injectate was indirectly validated by a cadaveric simulation, in which the dyed agent was injected into a cadaver emulating a living patient. Although there are obstacles to entirely depending on this method owing to its significant differences from real practice with a living object, it can provide reliable insight with other anatomical or histological evidence. Hence, we performed a histological inspection of the SSC to observe the intramuscular fascial septum and a cadaveric simulation to confirm whether there is a fascial barrier to prevent the spread.

A recent study demonstrated differences in the electromyography (EMG) activities of the superior and inferior compartments of the SSC^8^. The two compartments of the SSC would be selectively involved during various shoulder movements^5^, different effects on the SSC can result from BoNT injection on an individual partition of the muscle. Skeletal muscle consists of two types of fibers roughly classified according to function and oxygen demand: type I (slow-twitch) and type II (fast-twitch) fibers. Type I fibers support longer endurance activities but less force, with a more abundant blood supply, mitochondria, and myoglobin than do type II fibers. The histological composition of type I or II fibers should also be revealed to improve the effectiveness of the injection. Therefore, in this study, using immunohistochemistry with fresh cadavers, deliberate histological profiling was performed to determine which fibers are predominant within each compartment.

We aimed to verify the fascial compartmentation of the SSC and elucidate its histological components of type I and II fibers using macroscopic, histological observation and cadaveric simulation for providing an anatomical reference of efficient injection of the BoNT into the SSC.

## Materials and methods

### Study design and ethics statements

Seven fixed and three fresh cadavers (six males and four females; mean age, 82.5 years) were used in this study. All study procedures approved by the Surgical Anatomy Education Centre, Yonsei University College of Medicine (approval number: YSAEC: 23-005). The participants have provided informed consent to donate their bodies for research purposes. The authors state that every effort was made to follow all local and international ethical guidelines and law that pertain to the use of human cadaveric donors in anatomical research^9^. All specimens were harvested from the cadavers with no history of trauma or surgical approach in the subscapular region.

We carefully dissected the skin, subcutaneous tissue, deep fascia, and superficial vessels and identified the fascial septum of the SSC. The brachial plexus was examined in the axillary region to observe the extramuscular innervation pattern of the SSC. The nerve twigs that entered the SSC were thoroughly examined during or after dissection, and the extramuscular innervation patterns were accurately observed.

### Ultrasound-guided injection and serial sectional dissection

Two specimens were harvested from the two cadavers and used to evaluate SSC compartmentation. All specimens were placed in the supine position, and injections were performed by an experienced anesthesiologist. An in-plane technique with real-time ultrasound guidance was used to target the compartment. A TE7 ultrasonographic machine (Mindray Bio-Medical Electronics, Shenzhen, Guangdong, China) with a high-frequency linear probe (4–16 MHz) and an 80-mm, 22-gauge block needle were used to perform all injections. Once the needle tip just reached the superior compartment of the SSC, without piercing the fascial septum, its correct location was identified by injecting 1.0–2.0 mL of normal saline. After confirming the correct localization of the needle tip, 50 mL of 0.3% methylene blue solution was injected at a standard rate over 2 min. After the injection, the anatomist performed a serial sectional dissection.

### Application of the Sihler’s stain and analysis of innervation pattern of the subscapularis muscle

Five specimens were harvested from five cadavers, and the Sihler’s staining protocol was performed in seven steps as follows^4,10^:

1. The specimens were soaked in 10% unneutralized formalin for 1 month
2. The fixed specimens were soaked in 3% aqueous potassium hydroxide containing 3% hydrogen peroxide for 1 month
3. The macerated specimens were immersed in Sihler’s solution I (glacial acetic acid: glycerin: 1% aqueous solution of chloral hydrate = 1:1:6) for 1 month
4. The decalcified specimens were stored in Sihler’s solution II (Ehrich’s hematoxylin: glycerin: 1% aqueous solution of chloral hydrate = 1:1:6) for 1 month
5. The stained specimens were repeatedly placed in Sihler’s solution for a few hours to a few days, depending on the specimen size
6. The specimens were placed in 0.05% lithium carbonate solution for 1 h and washed in tap water for 1 h
7. The neutralized specimens were stored in a series of glycerin solutions daily for transparency.

Finally, the stained specimens were inspected using a medical film readout device to assess the nerve distribution. The intramuscular innervation pattern according to SSC compartmentation was closely analyzed.

### Analysis of the muscle component histological profile

To confirm the muscle type according to the region, the specimens were divided into three regions. Three fresh specimens were used for routine histological procedures and cut into 5-µm thick sections. The sections were immunohistochemically stained and Masson’s trichrome stained to compare muscle morphology. To identify the contributions of muscle types I and II to the SSC, the sections were incubated with primary antibodies against Monoclonal Anti-Myosin slow skeletal muscle antibody (mouse, diluted 1:500; Sigma-Aldrich, Inc., St. Louis, MO, USA) and Monoclonal Anti-Myosin fast skeletal muscle antibody (mouse, diluted 1:500; Sigma-Aldrich, Inc., St. Louis, MO, USA). Biotinylated secondary antibodies (Jackson ImmunoResearch, West Grove, PA, USA) were used to detect the primary antibodies. Subsequently, a streptavidin-tagged horseradish peroxidase kit (Vector Laboratories, Burlingame, CA, USA) was used to amplify the signal, and visualization of the antibody was performed using a NovaRED detection kit (Vector Laboratories, Burlingame, CA, USA). Mayer’s hematoxylin was used as a counterstain.

All sections were examined under an optical microscope (BX51; Olympus, Inc., Tokyo, Japan). Morphometric data are presented as a mean ± standard deviation (SD). Immunohistochemical staining revealed the density of each type of fiber compared with the whole muscle area in superior and inferior compartments of the SSC. The ratios of type I and type II muscles to the cross-sectional area were calculated in microns squared for individual cross-sectional images (ImageJ version 1.53n, NIH, USA). A parametric t-test was used because of the normal data distribution. All statistical analyses were performed using the Statistical Package (version 24.0; IBM Corporation, Armonk, NY, USA). A p value < 0.05 was considered significant.

## Results

**Figure 1:**
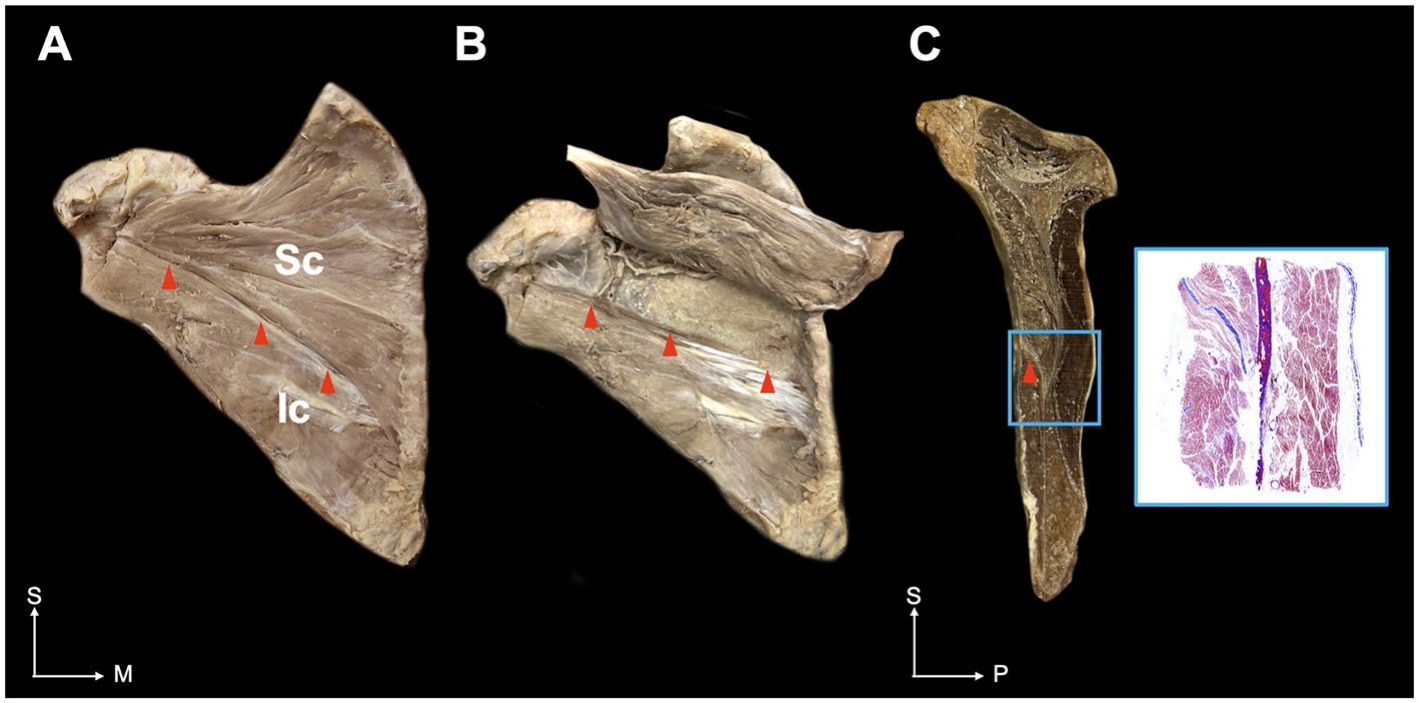
Fascial septum of the subscapularis (SSC) in the cadaver. The red dotted line indicates the fascial septum separating the superior compartment from the inferior compartment (**A**). The superior and inferior compartments are clearly divided by the fascial septum (**B**). In the sagittal section view, the fascial septum divides the superior and inferior compartments (**C**). The red arrowheads indicate the fascial septum. The blue box indicates the histological evaluation. *Sc* superior compartment of the SSC, *Ic* inferior compartment of the SSC, *S* superior, *M* medial.

The dissected specimens revealed a distinct fascia demarcating the SSC into the superior and inferior compartments (Fig. 1). On the anterior aspect of the SSC, the septum was shown as a line on the scapula from the glenoid (lateral end) to the vertebral side (medial end). In the sagittal section, the septum was attached to both the periosteum of the scapula and fascia of the SSC and was situated in the form of a bevel running forward. Histologically, a partitioned structure consisting of dense collagen tissue corresponding to the fascial septum was observed.

**Figure 2:**
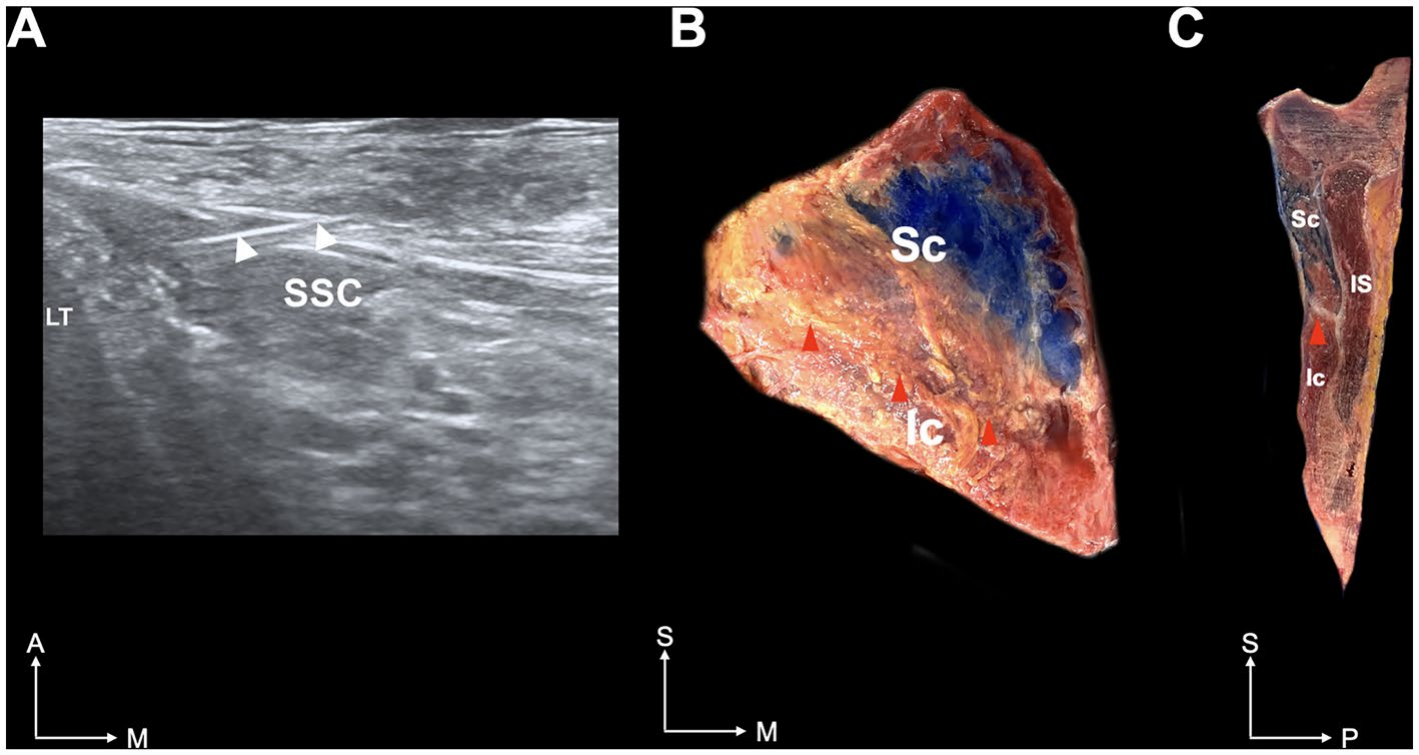
Ultrasound-guided image (**A**) of an injection into the subscapularis muscle (**B**) and sectional image (**C**) of the corresponding injected site. The ultrasound image demonstrates the needle placement (arrowheads). The blue dye is targeted to the superior compartment. The compartments are shown to be separated by the fascial septum. Red dotted line and red arrowhead indicate the fascial septum. *LT* lesser tubercle, *SSC* subscapularis muscle, *Sc* superior compartment of the SSC, *Ic* inferior compartment of the SSC; *IS* infraspinatus, *A* anterior, *M* medial, *S* superior, *P* posterior.

The superior compartment was a pan-shaped muscle situated in the upper two-thirds of the SSC with an apex inserted into the LT. The muscle fibers were arranged in a radial form that spread widely laterally. The inferior compartment was a rectangular muscle with a medial attachment to the LT in the lower one-third of the SSC. Muscle fibers in the inferior compartment were arranged parallel to the fascial septum. The medial attachments of the two compartments to the LT were located vertically to a similar extent. Our cadaveric simulation (Fig. 2), in which a dye solution was injected into the superior compartment, demonstrated that the agent could not penetrate the fascial septum.

**Figure 3:**
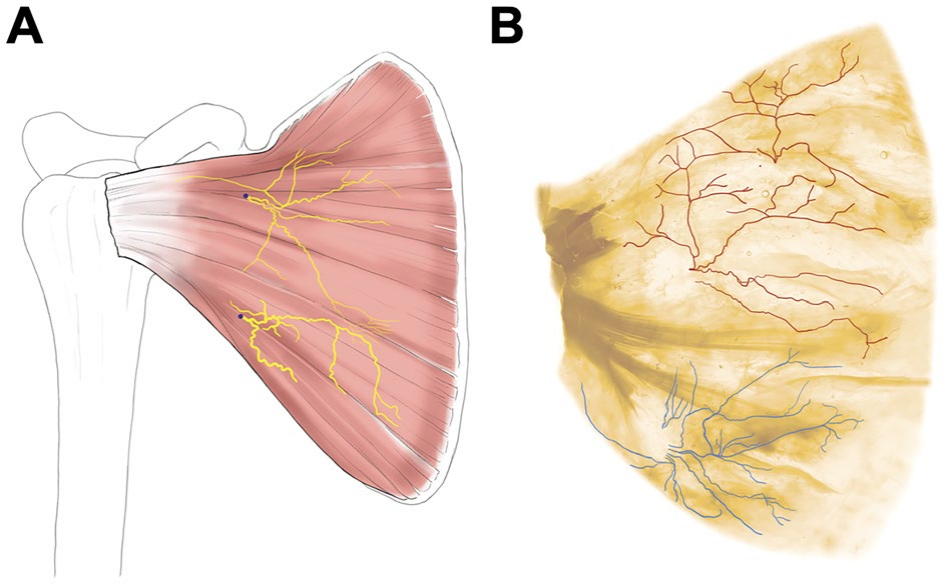
Neuromuscular compartmentation of the subscapularis muscle (SSC). Illustration (**A**) and Sihler’s stained specimen (**B**) of typical innervation pattern is shown (**A**). Main branches of the upper subscapular nerve (USN) and lower subscapular nerve (LSN) are identified to be compartmentalized; however, the tiny branches of the USN and LSN are widely distributed in the SSC. Red and blue lines indicate the USN and LSN, respectively.

The septum strongly restricted the agent’s flow into the inferior compartment. In addition, Sihler’s staining revealed that the USN and LSN innervated the SSC (Fig. 3), with two territories distributed by each nerve, mostly corresponding to the superior and inferior compartments of the muscle, although there were some tiny communicating twigs between the USN and LSN.

**Figure 4:**
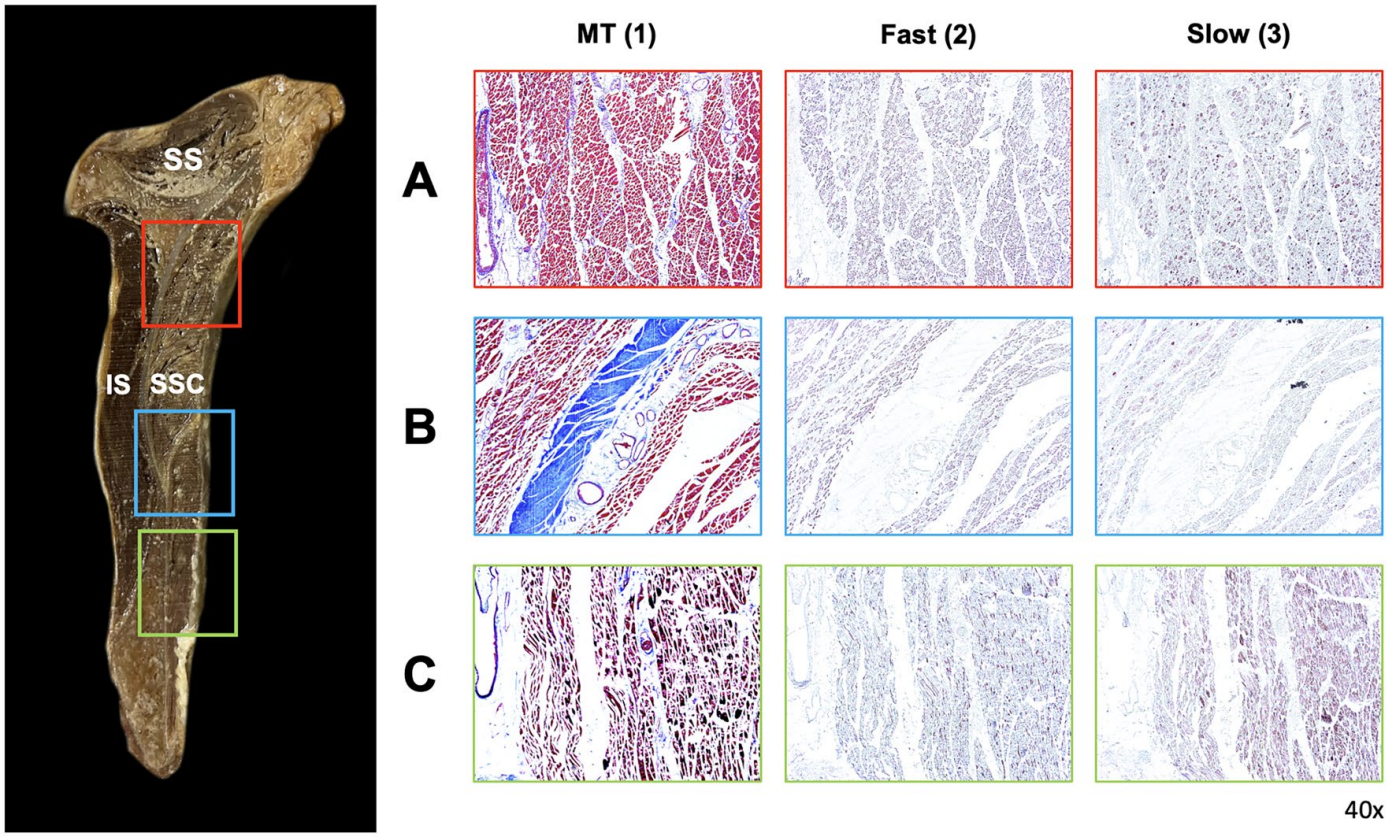
Analysis of the muscle component histological profile of the subscapularis muscle. The 40 × magnified image of the superior compartment (row A, red box), transitional area around the fascial septum (row B, blue box), and inferior compartment (row C, green box) of the SSC are observed by the Masson’s trichrome (column 1) and immunohistochemistry (columns 2 and 3). Columns 2 and 3 indicate the fast and slow type muscles, respectively.

Histomorphological inspection revealed the histological profiles of type I (slow-twitch) and II (fast-twitch) muscle fibers (Fig. 4).

**Figure 5:**
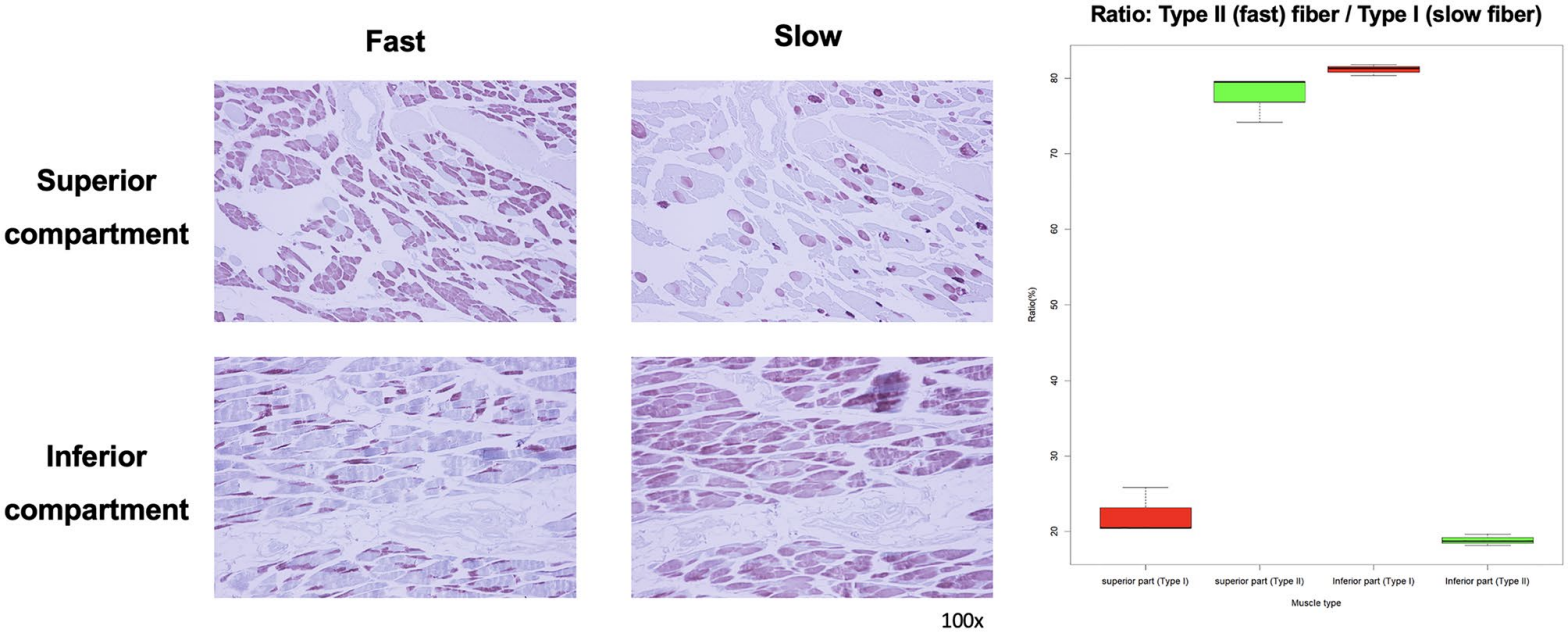
Regional immunoactivity ratio between the type I and type II muscles in the two regions (superior and inferior compartments) of the subscapularis muscle (SSC) presented in Fig. 4. Compared with the whole muscle area, the densities of the type I (slow-twitch) fibers were 22.26 ± 3.11% (mean ± standard deviation) in the superior and 81.15 ± 0.76% in the inferior compartments, and the densities of the type II (fast-twitch) fibers were 77.74% ± 3.11% in the superior and 18.85 ± 0.76% in the inferior compartments.

There was a difference in the fiber composition between the superior and inferior compartments of the SSC. The two compartments contained both type I and II fibers; however, the ratios of types I and II were relatively different. In the Mason trichrome-stained images, the cross-sectioned fibers accounted for 45.9% of the superior compartment and 43.58% of the inferior compartment. The immunohistochemical stain revealed the density of each type of fiber. Compared with the whole muscle area, the densities of the type I fibers were 22.26 ± 3.11% (mean ± SD) in the superior and 81.15 ± 0.76% in the inferior compartments, and the densities of the type II fiber were 77.74% ± 3.11% in the superior and 18.85 ± 0.76% in the inferior compartments (Fig. 5). The relative ratio in the superior compartment was lower in type I than in type II, and the ratio in the inferior compartment was lower in type II than in type I, with a statistically significant difference between the two types (p < 0.05) (Fig. 5). In summary, the superior compartment had more type II fibers than did the inferior compartment, and the ratio of type I was lower than that of type II.

## Discussion

Our histological and macroscopic inspections demonstrated typical compartmentation of the SSC by a physical partition of the fascial septum. The SSC was demarcated into larger superior and inferior compartments by a definite septum consisting of dense collagen tissues. The compartments nearly corresponded to two separate neuromuscular territories of the USN and LSN. All muscle compartments had both type I and type II muscle fibers; however, type II fibers were more abundant in the superior compartment, whereas the inferior compartment was mainly composed of type I fibers.

Neuromuscular segmentation of the SSC is important for determining optimal injection sites. Because the main routes of the two subscapular nerves are far away within this muscle, a small amount of BoNT injected from one site would insufficiently spread to the overall extent of the muscle. Previously, we reported an intramuscular innervation pattern of the SSC that could be segmented by the territories of USN and LSN, although some twigs formed a nervous connection between them. Similarly, Warden et al.^5^ reported neuromuscular partitioning of the SSC based on intramuscular innervation patterns, in which the SSC consisted of three (78%) or two (22%) partitions. We also found that the LSN was divided into two branches and had two independent entrances to the SSC. However, they proceeded closely with each other, and the fascial septum in the present study was located only between the two territories of the USN and LSN. No physical septa were observed between the LSN branches. Hence, partitioning the SSC into two compartments seems to be more clinically reliable for BoNT injections.

The present study is the first to demonstrate that the fascial septum can act as a physical barrier to obstruct the spread of BoNTs. This innervation pattern is widely believed to be essential for BoNT injection. However, the MEP is the actual site for the toxin molecule. Previous studies on BoNT injection using dissection or Sihler’s staining provided intramuscular innervation patterns based on the assumption that the MEPs were mainly located near the visible nerve endings. However, the physical condition for spreading the toxin has been overlooked as a determining factor for the efficiency of BoNTs. However, an intramuscular fascial structure should be seriously considered for BoNT injection because it can decrease the spread of BoNT by up to 23%^7^. Outcomes from BoNT injection to the SSC vary, ranging from significant improvement to no difference compared with placebo^2,3,11^. This discrepancy may arise from intramuscular innervation and the fascial septum identified in this study. Similarly, our cadaveric simulation showed that the agent injected into one compartment of the SSC did not penetrate the septum or spread to another. This strongly supports previous recommendations that multiple injections of BoNTs can be more effective than a single injection to only one site. Multiple injections can also fully overcome spasticity, even with significantly less BoNT than a single injection^5,6^. A physician must also be aware that overdiffusion of BoNT can result in weakness of the neighboring muscle^12^. Individual injections of BoNT into each compartment can be performed using an ultrasound image of the muscle, which will prevent weakness of neighboring muscles by decreasing the amount of the injectate.

The superior compartment of the SSC seems to contract more dynamically and immediately than the inferior compartment because of its higher proportion of type II fibers. Previous functional studies that made the approach medially to record EMG activity reported that the activity increased more in the superior compartment of the SSC during internal rotation and flexion of the arm, the major actions of the muscle, than in the inferior compartment^13,14^. Other studies with lateral approaches demonstrated that the EMG activity of the inferior compartment was higher than that of the superior compartment. However, the medial approach is more reliable for recording EMG signals because the SSC is triangular with a larger medial border. Furthermore, in the present study, the superior compartment of the muscle was significantly larger than the inferior compartment. Therefore, the superior and inferior compartments in previous studies using lateral approaches may belong to the inferior compartment in our study. O’Connor et al.^15^ reported that the superior compartment of the SSC generated a significantly earlier onset of EMG activity than did the inferior compartment. The results of these reliable EMG studies using a medial approach are consistent with those of ours, showing that the superior compartment with abundant type II fibers can provide a more powerful phasic force with a short duration than the inferior compartment.

The SSC also plays a role in stabilizing the glenohumeral joint as a compartment of the rotator cuff. It is the only rotator cuff muscle that internally rotates the humerus. In coordination with the rest of the rotator cuff, the SSC provides active stabilization of the glenohumeral joint during the external rotation and flexion of the arm. This muscle offers reciprocal opposite action against the external rotation of the arm by the rest to maintain a balanced articulation between the humeral head and glenoid fossa of the scapula^8^. The lower portion of the SSC also resists anterior luxation of the joint by its tonic contraction, passively contributing to stabilizing the glenohumeral joint. It is difficult to conclude which compartment plays a more significant role in tonic contraction for stabilization based only on the histological profiling of the muscle type. However, the inferior compartment of the SSC would be supposed to provide resistant tonic contractions for a long duration for active and passive stabilization of the glenohumeral joint. This seems to be because of the abundance of type I muscle fibers in the inferior compartment.

Type I muscle fibers, also known as red muscle fibers, are more aerobic than type II fibers. They require sufficient blood supply as a tissue with abundant mitochondria and myoglobin to meet their exuberant oxygen demand. Previously, we delineated the arterial distribution to the SSC and reported that the subscapular and lateral thoracic arteries from the axillary artery were mainly supplied to the muscle, with supplementary distribution by the subclavian artery branches. The area near the inferior border of the SSC receives sufficient blood from the subscapular, lateral thoracic, or posterior circumflex humeral arteries. By contrast, the suprascapular artery from the subclavian artery is supplied to the superior margin. Unlike the innervation pattern, the distinct boundaries of each territory supplied by individual arteries are difficult to identify owing to their ambiguity. Nonetheless, it can be hypothesized that the blood supply to the inferior compartment of the SSC is not less than that to the superior compartment. In the present study, this anatomical environment with rich arterial distribution fully meets the oxygen demand of type I fibers in the inferior compartment of the SSC muscle.

This study has a couple of limitations. The most significant limitation is that we harvested fresh muscle tissue from older cadavers. Pingel et al.^16^ noted that sarcopenia due to disuse by aging could result in a fast-slow fiber-type shift. Thus, it is difficult to verify the actual muscle type for all ages or to decide whether a muscle compartment is more engaged in tonic or phasic action based on the proportion of slow-fast muscle fibers, even if our histological profiling can specify exactly the muscle type. However, a tendency of a slow-fast fiber proportion was consistently observed without significant discrepancies among the samples. Rather, it seems to support that the SSC plays a role in active and passive stabilization, even in older individuals. Physiological examination using EMG with ultrasound images is required to prove regional differences in SSC muscle function resulting from physical compartmentation. In addition, clinical trials should be conducted to demonstrate that the fascial septum affects BoNT efficiency.

## Conclusion

In conclusion, this study comprehensively used various methods, including gross observation, histological inspection, and cadaveric simulation with ultrasound images, and successfully demonstrated that the SSC is partitioned into two compartments by the fascial septum, which can act as a powerful physical barrier against BoNT spread. Furthermore, the compartments had different proportions of slow-fast muscle fibers, corresponding to the functional differences between the superior compartment as an early-onset internal rotator and the inferior compartment as a durable stabilizer of the glenohumeral joint.

## Data Availability

The datasets used and/or analysed during the current study available from the corresponding author on reasonable request.

## Abbreviations

SSC: Subscapularis muscle
LT: Lesser tubercle
BoNT: Botulinum neurotoxin
MEP: Motor end plate
EMG: Electromyography
SD: Standard deviation
USN: Upper subscapular nerve
LSN: Lower subscapular nerve
